# Construction and validation of the Emotional Trust in Artificial Intelligence Scale (CEIA)

**DOI:** 10.3389/fpsyg.2026.1755160

**Published:** 2026-03-12

**Authors:** Berle Estalin Briones-Llamoctanta, Ronald Garnique Hinostroza, Roberto Estrada-Medina, Josué Edison Turpo-Chaparro

**Affiliations:** 1 Escuela Profesional de Psicología, Universidad Peruana Unión, Lima, Peru; 2 Escuela de Posgrado, Universidad Peruana Unión, Lima, Peru

**Keywords:** artificial intelligence, emotional trust, factor analysis, human-AI interaction, measurement invariance, psychometric validation

## Abstract

**Background:**

Emotional trust in Artificial Intelligence (AI) has become a crucial component of human-AI interaction, especially in academia, where generative systems are used for academic, communicational, and socio-emotional purposes. While research has advanced in cognitive trust, there is a lack of validated instruments that rigorously assess the affective dimension of trust, which involves processes of perceived authenticity, emotional validation, affective recognition, and the establishment of symbolic bonds with artificial agents.

**Objective:**

To develop and psychometrically validate the Emotional Confidence in Artificial Intelligence Scale (CEIA) in university students, examining its factor structure, reliability, convergent and discriminant validity, as well as its measurement invariance by sex.

**Methods:**

Six hundred and fibe Peruvian university students, aged 16 to 88 years (M = 22.93, SD = 7.35), participated in the study. Item distributions, item-total correlations, and descriptive statistics were analyzed. Internal structure was assessed using Exploratory Factor Analysis (EFA) with polychoric correlations and Confirmatory Factor Analysis (CFA). Reliability was estimated using Cronbach's alpha and McDonald's omega. Convergent and discriminant validity were examined using AVE, CR, and HTMT. Sex invariance was assessed using multigroup CFA with the WLSMV estimator.

**Results:**

The EFA supported a multidimensional structure consistent with the theoretical model. The CFA confirmed a four-factor solution—Perceived Authenticity, Emotional Validation, Affective Recognition, and Symbolic Bonding—with adequate fit indices (CFI = 0.944; TLI = 0.937; RMSEA = 0.073; SRMR = 0.033). The dimensions showed high internal consistency (α = 0.91–0.94; ω = 0.91–0.94) and satisfactory evidence of convergent and discriminant validity. Invariance analysis demonstrated metric invariance and partial scalar invariance between men and women.

**Conclusion:**

The CEIA is a valid, reliable, and partially sex-invariant instrument for assessing emotional trust in AI among university students. It constitutes a robust tool for research, technological design, and the ethical development of AI systems with affective capabilities.

## 1 Introduction

The rapid expansion of generative artificial intelligence (AI) has transformed human interactions in multiple spheres of daily, academic, and professional life. From conversational tools like ChatGPT (OpenAI, San Francisco, CA, United States), Copilot (Microsoft Corporation, Redmond, WA, United States), and Gemini (Google LLC, Mountain View, CA, United States) to virtual agents with psychological support functions, AI has ceased to be a merely technical instrument and has become a symbolic and emotional interlocutor in people's lives (Huynh and Aichner, 2025; Volpato et al., 2025). This transformation has sparked a growing interest in understanding the nature of the trust users place in systems that, while not human, exhibit empathetic, communicative, and seemingly understanding behaviors (Riley and Dixon, 2024). Consequently, trust in AI can no longer be reduced to a cognitive phenomenon of evaluating competence, transparency, or algorithmic security, but must also be conceived as an affective and relational experience (Bhat, 2025; Yao et al., 2024).

Several recent studies suggest that users' emotional responses to AI are stronger predictors of trust and technological acceptance than rational judgments of utility or performance (Kwilinski et al., 2025; Sun et al., 2024). This shift toward the emotional raises new forms of symbolic connection with artificial agents, where the perception of empathy, understanding, or interpersonal warmth becomes a key component of the interaction (Dorton and Harper, 2022). In educational and university settings, this trend is particularly relevant, as students use chatbots not only for academic purposes but also, as spaces for emotional release or personal consultation (Chin et al., 2023; Torres-Gastelú and Torres-Real, 2025). This emerging form of AI-mediated emotional interaction could modify the dynamics of trust, support, and technological dependence (Morales-García et al., 2024; Ta et al., 2020), making it necessary to develop instruments capable of accurately assessing the emotional dimension of such trust.

In this context, emotional trust in artificial intelligence (ETAI) is defined as the degree to which a person attributes to an artificial agent the capacity to understand, validate, and respond to their affective states in a coherent, authentic, and meaningful way, generating a perception of symbolic interpersonal connection (Jiang et al., 2025; Kolomaznik et al., 2024; Shang et al., 2024). Based on the distinction between cognitive and affective trust in human-AI interaction, this construct is organized around four interrelated theoretical dimensions. Perceived authenticity, grounded in Nass and Moon's (2000) social media response theory, refers to the extent to which the user perceives truthfulness or sincerity in the AI's responses. Emotional validation, inspired by Rogers (1961) humanistic theory, is manifested when the user experiences acceptance and empathic understanding toward a non-human system. Affective recognition, derived from the theory of technological anthropomorphism (Epley et al., 2007), describes the attribution of emotions or intentions to agents capable of detecting or reflecting human emotional states. Finally, the symbolic bond, grounded in the theory of parasocial interaction (Horton and Wohl, 1956), explains how individuals can develop unidirectional emotional ties with digital entities that simulate affective reciprocity. Taken together, these dimensions reflect an emerging psychological process of the digital age: the extension of attachment and emotional trust to AI systems capable of emulating empathy and emotional containment (Liu et al., 2024).

In recent years, research on trust in AI has progressed toward the creation of more refined and culturally adaptable psychometric instruments. McGrath et al. (2025) The Trust in Automation Scale (TIAS) and its abbreviated version (S-TIAS) were validated, demonstrating that trust in AI can be accurately measured using brief, highly sensitive items. Similarly, Wang et al. (2025) a Generative Human-AI Trust Scale was developed, composed of three dimensions: benevolence, competence, and reciprocity, confirming that trust is a multifaceted construct essential for technological acceptance. Thus, McGrath et al. (2025) address trust in AI from a rational and functional perspective, and Wang et al. (2025) consider it a predictor of technological acceptance, while the emotional and relational perspective, considering it a possible indicator of wellbeing and affective health in human-AI interaction, remains largely unexplored. Along the same lines, other studies (Perrig et al., 2023; Visser et al., 2025) warn that traditional measures tend to confuse trust with dependence or mistrust, highlighting the need for conceptually clearer instruments that distinguish between rational, emotional, and behavioral trust in intelligent systems.

In parallel, approaches focused on the user's affective experience with generative AI are emerging. Montag and Elhai (2025) The AI Interaction Positivity Scale (AI-IPS) was proposed, demonstrating a positive correlation between life satisfaction and trust in tools like ChatGPT, suggesting that emotional interaction directly influences technological acceptance. Similarly, Martín-Moncunill et al. (2024) the Perceived Operational Trust Design in Artificial Intelligence (POTDA9I) tool was presented, designed to measure the degree of perceived operational trust, while Alonso Moral (2025) emphasized that explainability (XAI) is an ethical prerequisite for the reliability of intelligent systems. These advances reflect a transition from purely cognitive trust to emotional and symbolic trust, where users attribute intentions, empathy, and authenticity to artificial agents, reinforcing the need for evaluation frameworks that integrate the affective dimension into the human-AI relationship.

In this context of rapid conceptual and methodological evolution, the lack of instruments that capture emotional trust in AI from a comprehensive and cross-cultural perspective is evident. Existing scales address the reliability or technical competence of the system, but neglect the affective dimension that underpins the symbolic bond between humans and artificial agents. Given that interaction with generative technologies such as ChatGPT or Copilot, and others, transcends the functional to involve processes of attachment, emotional validation, and affective recognition, it becomes imperative to develop measures that assess this new psychosocial phenomenon. Thus, this study proposes to design and validate the Emotional Trust in Artificial Intelligence Scale (CEIA), integrating the dimensions of perceived authenticity, emotional validation, affective recognition, and symbolic bond, and providing a specific measure for studying the affective human-AI relationship.

## 2 Methods

### 2.1 Design and participants

This study employed an instrumental design aimed at the psychometric validation of the Emotional Confidence in Artificial Intelligence Scale (CEIA), following the guidelines proposed by Ato et al. (2013) for the development and analysis of measurement instruments. Participants were recruited through non-probability convenience sampling, with the objective of obtaining a sufficiently large sample of university students who had regular exposure to generative artificial intelligence tools. Although the sample was drawn from a single university and exhibited limited institutional and disciplinary diversity, this characteristic is acknowledged and addressed as a limitation regarding the generalizability of the findings.

A total of 605 Peruvian university students participated in the study. The sample size was justified using multiple methodologically robust criteria for scale validation. First, the ratio of approximately 25 participants per item substantially exceeds the conservative recommendation of 10:1 proposed by Nunnally and Bernstein (1994). Second, according to Comrey and Lee's (1992) criteria, the sample size is classified as “excellent” (*N* > 500) for factor analytic procedures. Third, simulation studies indicate that this sample size provides statistical power greater than 0.95 for detecting adequately fitting confirmatory factor models (MacCallum et al., 1999). Finally, the sample exceeds international psychometric validation standards established by the COSMIN consortium (Mokkink et al., 2010).

To enhance the robustness and replicability of the factor structure, the total sample (*N* = 605) was randomly divided into two independent subsamples. The first group (*n* = 250) was used for exploratory factor analysis (EFA), as this procedure requires data unconditioned by prior model specifications. The second group (*n* = 355) was used for confirmatory factor analysis (CFA), ensuring independence between exploratory and confirmatory procedures and reducing the risk of capitalization on chance (VandenBos, 2015).

Participants ranged in age from 16 to 88 years (M = 22.93, SD = 7.35); 62.81% identified as male and 37.19% as female. All participants resided in Peru and reported prior experience using generative AI tools such as ChatGPT, Gemini, or Copilot. Cases with inconsistent demographic information, duplicate responses, or lack of informed consent were excluded. Detailed sociodemographic characteristics are presented in Table 1.

**Table 1:** Sociodemographic characteristics of the participants (*N* = 605).

**Variable**	**Category**	***n***	**%**
Sex	Female	225	37.19
	Male	380	62.81
Marital status	Single	521	86.1
	Married	53	8.7
	Divorced	21	3.5
	Cohabitant	10	1.6
Frequency of AI use	Diary	114	18.8
	Several times/week	213	35.2
	Occasional	258	42.6
	Never	20	3.3
Purpose of using AI	Studies	345	57.0
	Investigation	127	21.0
	Emotional support	78	12.9
	Others	55	9.1

Percentages calculated on *n* = 605.

### 2.2 Instrument

The Emotional Confidence in Artificial Intelligence, was developed using a multidisciplinary conceptual framework that integrates contributions from social psychology, human-computer interaction, AI-mediated communication, and symbolic attachment theory in digital environments. The ETAI is defined as the degree to which a person perceives that an artificial agent can understand, validate, and respond coherently and meaningfully to their affective states, generating experiences of simulated interpersonal connection (Jiang et al., 2025; Kolomaznik et al., 2024; Shang et al., 2024).

The construct is organized into four theoretical dimensions. Perceived authenticity is based on the social media response model (Nass and Moon, 2000), which proposes that individuals apply social norms even when interacting with machines. Emotional validation incorporates the Rogerian principle of empathic acceptance (Rogers, 2012), indicating that users can feel emotional understanding even from non-human agents. Affective recognition is based on the theory of technological anthropomorphism (Epley et al., 2007), which explains the attribution of emotions and intentionality to artificial systems. Finally, symbolic bonding is based on the theory of parasocial relationships (Horton and Wohl, 1956), which describes unidirectional emotional connections with digital entities that simulate reciprocity.

The 24 items were evaluated by five expert judges in psychology, digital communication, and artificial intelligence. Aiken's V coefficient was greater than 0.85 for all items and dimensions, with 95% confidence intervals, confirming excellent clarity, relevance, and conceptual representativeness (Aiken, 1980). The equations should be inserted in editable format from the equation editor.

### 2.3 Procedure

Participants were informed about the study's objectives, the voluntary nature of their participation, and the confidentiality of their responses. The questionnaire was administered digitally using electronic forms. Automated filters and manual review were implemented to ensure data quality, removing incomplete or inconsistent records.

#### 2.3.1 Data analysis

The analyses were performed in RStudio [Posit Software, PBC (formerly RStudio), Boston, MA, United States] (R Core Team, 2023) using the psych [Developed by William Revelle, Northwestern University, Evanston, IL, United States (CRAN, R Foundation for Statistical Computing, Vienna, Austria)], lavaan [Developed by Yves Rosseel, Ghent University, Ghent, Belgium (CRAN, R Foundation for Statistical Computing, Vienna, Austria)], and semTools packages (Developed by Jorgensen et al., CRAN, R Foundation for Statistical Computing, Vienna, Austria). First, descriptive statistics and corrected item-total correlations were calculated, and basic psychometric assumptions were verified. Skewness and kurtosis values within the ±1.5 range were considered acceptable (Finney, 2013; Kline, 2016).

The exploratory factor analysis for Group 1 (*n* = 250) employed polychoric correlations, MINRES extraction, and oblimin rotation, following the recommendations for ordinal items and correlated factors (Flora and Curran, 2004). Factor adequacy was verified using the KMO index and Bartlett's test of sphericity. The optimal number of factors was determined through parallel analysis.

Confirmatory factor analysis was performed with Group 2 (*n* = 355) using the MLR estimator, which is robust to deviations from normality. The fit indices examined were CFI, TLI, RMSEA with its 90% confidence interval, and SRMR (Schumacker and Lomax, 2015). Reliability was assessed using Cronbach's alpha and McDonald's omega coefficients (McDonald, 2013).

Convergent validity was analyzed using the average variance extracted (AVE), with values greater than 0.50 considered acceptable Fornell and Larcker, (1981). Discriminant validity was verified using the HTMT ratio following the criteria of Henseler et al. (2014).

Finally, factorial invariance by sex was examined using a multigroup CFA with the WLSMV estimator. Configural, metric, and scalar models were evaluated sequentially, and differences of CFI < 0.010 and RMSEA < 0.015 were considered evidence of invariance (Chen, 2007).

## 3 Results

The content validity of the instrument was assessed using Aiken's V coefficient with the participation of five expert judges in psychometrics, artificial intelligence, and psychological assessment. Each judge rated the relevance, clarity, and coherence of the 24 items and the four proposed dimensions. The results revealed V values ranging from 0.88 to 1.00, significantly exceeding the recommended minimum cutoff point of 0.70 for acceptable validity and 0.80 for excellent validity (Aiken, 1980; Penfield and Giacobbi, 2004). Furthermore, the 95% confidence intervals confirmed that all items achieved values significantly above the critical level, demonstrating systematic agreement among the judges regarding the conceptual and wording quality of the instrument. Similarly, the four dimensions obtained V values above 0.90, supporting their theoretical relevance and their suitability for representing the core components of emotional trust in artificial intelligence. Overall, these results indicate that the CEIA possesses robust and consistent content validity, making it suitable for use in psychological research and assessment contexts.

### 3.1 Sociodemographic characteristics of the sample

The final sample consisted of 605 Peruvian university students from various academic fields. Regarding gender, 62.81% identified as male and 37.19% as female. As for marital status, the majority of participants were single (86.1%), followed by smaller percentages of married (8.7%), divorced (3.5%), and cohabiting (1.6%), which is consistent with the typical profile of a university population. Regarding the use of artificial intelligence tools, the vast majority reported using them with some regularity: 42.6% used them occasionally, 35.2% several times a week, and 18.8% daily, while only 3.3% indicated not using them. The main purpose reported was academic (57.0%; e.g., homework and studying), followed by research and writing activities (21.0%), emotional support or conversation (12.9%), and other uses (9.1%). Overall, these results show that the student sample maintains frequent contact with generative AI systems, both academically and emotionally, providing a relevant context for assessing emotional trust in these systems (see Table 1).

### 3.2 Descriptive statistics of the items

Table 2 presents the descriptive statistics for the 24 items of the Emotional Confidence in Artificial Intelligence Scale (ECAI). Means ranged from 2.90 to 3.26, indicating a moderate tendency toward intermediate and high agreement levels on the five-point Likert scale. Standard deviations were around 1.0, demonstrating adequate variability and the absence of extreme ceilings or floors. The item distributions showed skewness and kurtosis values within the ranges considered acceptable for factor analyses with large samples (approximately ±1.5, Kline, 2016). Skewness was predominantly negative and of small magnitude, reflecting a slight tendency toward more favorable responses regarding emotional trust in AI. Similarly, kurtosis remained close to mesokurtosis, with no indication of excessive tailing. These results suggest that, although the data are ordinal, their empirical behavior is compatible with assumptions of approximate normality that facilitate factor modeling (see Table 2).

**Table 2:** Descriptive statistics of the CEIA items (*N* = 605).

**Item**	**M**	**OF**	**Min-Max**	**Asymmetry**	**Kurtosis**
AP1	3.22	1.01	1–5	−0.62	−0.21
AP2	3.18	0.94	1–5	−0.31	−0.38
AP3	3.26	0.95	1–5	−0.45	−0.15
AP4	3.21	0.96	1–5	−0.42	−0.22
AP5	3.26	0.97	1–5	−0.42	−0.28
AP6	3.15	0.94	1–5	−0.32	−0.26
VE1	3.08	0.99	1–5	−0.28	−0.27
VE2	3.09	1.00	1–5	−0.31	−0.41
VE3	3.03	1.00	1–5	−0.30	−0.36
VE4	3.08	1.02	1–5	−0.37	−0.44
VE5	3.02	1.01	1–5	−0.30	−0.49
VE6	3.05	0.99	1–5	−0.35	−0.33
RA1	3.03	0.98	1–5	−0.25	−0.41
RA2	2.92	1.00	1–5	−0.20	−0.49
RA3	2.90	0.97	1–5	−0.20	−0.41
RA4	3.02	0.99	1–5	−0.26	−0.35
RA5	2.93	1.01	1–5	−0.24	−0.58
RA6	3.01	0.99	1–5	−0.29	−0.40
VS1	2.93	1.01	1–5	−0.23	−0.51
VS2	2.98	1.02	1–5	−0.30	−0.47
VS3	2.96	1.03	1–5	−0.25	−0.52
VS4	2.98	1.03	1–5	−0.27	−0.48
VS5	2.94	0.99	1–5	−0.29	−0.41
VS6	3.05	1.02	1–5	−0.28	−0.45

AP, Perceived Authenticity; VE, Emotional Validation; RA, Affective Recognition; VS, Symbolic Bond.

### 3.3 Internal reliability

The internal consistency of the CEIA was assessed using Cronbach's alpha coefficient (α) and McDonald's omega coefficient (ω), both at the subscale level and for the total scale. As shown in Table 3, the four theoretical dimensions—Perceived Authenticity (PA), Emotional Validation (EV), Affective Recognition (AR), and Symbolic Bonding (SB)—achieved very high coefficients. Specifically, alpha values ranged from 0.91 to 0.94, while omega values were in a similar range (0.91–0.94). These levels significantly exceed the conventional criterion of 0.70 and fall within the range considered excellent reliability (Kline, 2016), indicating high internal homogeneity of the items within each dimension.

**Table 3:** Reliability coefficients by dimension and total scale.

**Dimension**	**α**	**ω**	**BIRD**
Perceived Authenticity (PA)	0.913	0.913	0.637
Emotional Validation (EV)	0.939	0.940	0.722
Affective Recognition (AR)	0.936	0.937	0.713
Symbolic link (VS)	0.936	0.937	0.712
Full scale	0.975	0.981	–

The AVE corresponds to the average variance extracted for convergent evaluation.

The total scale showed an α = 0.975 and a ω = 0.981, highlighting the presence of a strong overall emotional trust factor in AI, in addition to the four specific dimensions. This evidence supports the use of both subscale and global scores for the CEIA (see Table 3).

### 3.4 Evidence of validity related to internal structure: exploratory factor analysis

To examine the internal structure of the Emotional Confidence in Artificial Intelligence Scale (CEIA), an Exploratory Factor Analysis (EFA) was performed using the polychoric correlation matrix, consistent with the ordinal nature of the items (Flora and Curran, 2004). Sample adequacy was excellent, with a KMO index of 0.94, clearly exceeding the 0.80 criterion required for stable factor solutions. Furthermore, all measures of individual adequacy (MSA) exceeded 0.89. Bartlett's test of sphericity was significant (χ^2^ = 18,266.43, df = 276, *p* < 0.001), confirming the appropriateness of conducting a factor analysis.

**Figure 1:**
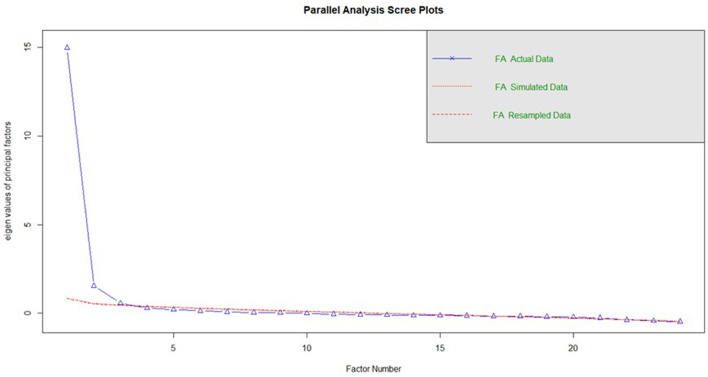
Parallel analysis.

The optimal number of factors was determined using polychoric parallel analysis (Figure 1), the results of which showed that only the first two actual eigenvalues exceeded the simulated ones. This pattern suggests an initial structure with two common sources of variance.

However, the CEIA is theoretically based on four distinct affective components—Perceived Authenticity, Emotional Validation, Affective Recognition, and Symbolic Bonding—and it is recognized that, in emotional scales with highly correlated constructs, the factors can be grouped in exploratory solutions (Brown, 2015; Fabrigar et al., 1999). Indeed, the Affective Recognition and Symbolic Bonding items showed high correlations, which led to both constructs emerging as a single factor in the initial solution.

Considering this empirical convergence and the scale's conceptual structure, a three-factor model was estimated using the MINRES method with oblimin rotation (Table 4). This solution explained 61.1% of the total variance and showed high and well-defined factor loadings (>0.60), without the need to remove items. The identified factors corresponded to: (a) a joint RA–VS factor, (b) Emotional Validation (EV), and (c) Perceived Authenticity (PA). Overall, the EFA suggests an empirical three- dimensional structure with strong internal consistency, which will be subsequently tested through confirmatory analyses.

**Table 4:** Factor loadings of Exploratory Factor Analysis (EFA).

**Item**	**MR1**	**MR2**	**MR3**
AP1	–	0.674	–
AP2	–	0.816	–
AP3	–	0.883	–
AP4	–	0.773	–
AP5	–	0.762	–
AP6	–	0.433	0.543
VE1	–	–	0.862
VE2	–	–	0.641
VE3	0.321	–	0.569
VE4	–	–	0.666
VE5	–	–	0.728
VE6	–	–	0.606
RA1	0.567	–	–
RA2	0.677	–	–
RA3	0.711	–	–
RA4	0.818	–	–
RA5	0.804	–	–
RA6	0.682	–	–
VS1	0.801	–	–
VS2	0.764	–	–
VS3	0.978	–	–
VS4	0.948	–	–
VS5	0.917	–	–
VS6	0.785	–	–

Exploratory factor analysis was performed on the polychoric correlation matrix using the MINRES method and oblimin rotation, in accordance with the ordinal nature of the items. Three factors were retained based on parallel analysis and theoretical criteria, explaining 61.1% of the total variance. The factors correspond to Affective Recognition/Symbolic Bonding (RA–VS), Emotional Validation (VE), and Perceived Authenticity (AP).

### 3.5 Confirmatory factor analysis and model fitting

Based on the solution derived from the exploratory analysis, a confirmatory factor analysis (CFA) model was specified with four correlated factors corresponding to the theoretical dimensions of Perceived Authenticity, Emotional Validation, Affective Recognition, and Symbolic Bonding. The model was estimated using the robust MLR estimator, appropriate for ordinal items and for data that may deviate from multivariate normality.

The overall fit indices indicated a good model fit. Although the χ^2^_(246)_ statistic = 714.13 was significant, this is expected in large samples. Therefore, the evaluation focused on relative fit indices and approximate error. The CFI = 0.944 and TLI = 0.937 fell within the acceptable to good fit range (Hu and Bentler, 1999). The SRMR = 0.033 reflected an excellent fit in terms of residual discrepancy, and the RMSEA = 0.073 (90% CI = 0.067–0.080) was within the range considered adequate for models with a considerable number of items and a moderately complex latent structure.

The standardized factor loadings were all high and statistically significant (λ > 0.74), indicating that each item contributes substantially to its corresponding latent dimension. Taken together, these results support the proposed four-dimensional structure for the CEIA and provide strong evidence of structural validity (see Table 5).

**Table 5:** AFC goodness of fit indices.

**Index**	**Worth**
χ^2^ (df = 246)	714.13
CFI	0.944
TLI	0.937
SRMR	0.033
RMSEA	0.073
90% CI RMSEA	0.067 −0.080

The AFC supports the four-dimensional structure of the CEIA and demonstrates structural stability.

### 3.6 Assessment of common method bias

Because all measures were collected via self-report, potential common method bias (CMB) was examined. A single-factor CFA model showed substantially poorer fit (CFI = 0.893; TLI = 0.883; RMSEA = 0.191; SRMR = 0.070) compared with the theorized four-factor model (CFI = 0.966; TLI = 0.962; RMSEA = 0.109; SRMR = 0.032), suggesting that a single common factor does not account for the covariance among CEIA items. In addition, including a common latent factor (CLF) did not meaningfully alter substantive standardized loadings (median Δλ = 0.017; max Δλ = 0.068), supporting that common method variance is unlikely to be the primary driver of the CEIA factor structure.

### 3.7 Convergent and discriminant validity

Convergent validity was assessed using the average variance extracted (AVE) and the composite reliability of each dimension. AVE values ranged from 0.64 to 0.72, exceeding the 0.50 criterion established by (Fornell and Larcker 1981), indicating that each factor explains more than 50% of the variance in its indicators. Similarly, the composite reliability coefficients (ω and CR) ranged from 0.91 to 0.94, demonstrating high internal consistency for each subscale. Taken together, these results strongly support the convergent validity of the four dimensions of the CEIA.

Discriminant validity was examined using the heterotrait-monotrait (HTMT) index, calculated from the standardized correlations of the CFA model. HTMT values ranged from 0.74 to 0.95, an expected range given the highly interrelated nature of the affective processes assessed. Although several coefficients exceeded the strict threshold of 0.85, all remained below the 0.95 limit, considered adequate for conceptually close constructs or components of the same affective system (Henseler et al., 2015). This pattern suggests the presence of a robust general factor of emotional trust, accompanied by dimensions that retain sufficient conceptual differentiation to be interpreted as specific subdomains within the CEIA. Overall, the findings support acceptable discriminant validity, consistent with the proposed theoretical framework (see Table 6).

**Table 6:** HTMT matrix between the dimensions of the CEIA.

**Dimension**	**AP**	**VE**	**RA**	**VS**
AP	1	0.895	0.787	0.739
VE	–	1	0.916	0.878
RA	–	–	1	0.951
VS	–	–	–	1

HTML < 0.90 is acceptable; < 0.95 is permissible for related constructs.

### 3.8 Assessment of common method bias

The factorial invariance of the CEIA by sex was assessed using multigroup CFA with the WLSMV estimator, sequentially testing the configural, metric, and scalar models. The configural model showed a good overall fit (CFI_scaled = 0.967; RMSEA_scaled = 0.114; SRMR = 0.045), indicating that the four-dimensional structure of the scale is equivalent in men and women.

By restricting the factor loadings (metric model), the fit remained virtually unchanged (CFI_scaled = 0.968; RMSEA_scaled = 0.110; SRMR = 0.053) and the changes were less than the recommended criteria (ΔCFI = 0.001; ΔRMSEA = 0.004), which supports the metric invariance between groups.

In the scaled model, where joint restrictions were imposed on loads and thresholds, a further improvement in the scaled CFI (0.974) and a reduction in the scaled RMSEA (0.094) were observed. The change in CFI remained within the cutoff point (ΔCFI = 0.006), while the ΔRMSEA (0.017) slightly exceeded the criterion of 0.015. Taken together, these results suggest evidence of approximate or partial scalar invariance, so comparisons of latent means between men and women can be made, although they should be interpreted with some caution (see Table 7).

**Table 7:** Fit indices for sex invariance models.

**Model**	**CFI**	**ΔCFI**	**RMSEA**	**ΔRMSEA**
Configurable	0.967	–	0.114	–
Metric	0.968	−0.001	0.110	−0.004
Climb	0.974	−0.006	0.094	−0.016

### 3.9 Intercorrelations among CEIA dimensions

Finally, correlations between the mean scores of the four CEIA dimensions were analyzed in the total sample. All correlations were positive, of high magnitude, and statistically significant (*p* < 0.001). The strongest associations were observed between Emotional Validation and Affective Recognition (*r* = 0.853), as well as between Affective Recognition and Symbolic Bonding (*r* = 0.897), reflecting a high interrelationship between processes of emotional resonance, affective recognition, and parasocial bonding with AI agents. The Perceived Authenticity dimension also showed high correlations with the other subscales (*r* = 0.646–0.769), although slightly lower in magnitude. This correlational pattern is consistent with the conceptualization of emotional trust in AI as a set of closely linked affective processes, including the perception of agent authenticity, the experience of emotional validation, the recognition of affective sensitivity, and the development of symbolic bonds. The high correlations suggest the presence of an underlying general psychological component, while also supporting the usefulness of retaining the four theoretical dimensions that capture distinct nuances within the overall construct (see Table 8).

**Table 8:** Correlations between dimensions (*N* = 605).

**Dimension**	**AP**	**VE**	**RA**	**VS**
AP	1	0.769^***^	0.684^***^	0.646^***^
VE	–	1	0.853^***^	0.835^***^
RA	–	–	1	0.897^***^
VS	–	–	–	1

^***^p < 0.001.

## 4 Discussion

The objective of this study was to construct and validate the Emotional Trust in Artificial Intelligence Scale (CEIA) in a large sample of Peruvian university students, providing an instrument specifically oriented toward the affective dimension of trust in generative AI systems within a higher education context. The psychometric results obtained—consistent internal structure, high internal consistency, adequate evidence of convergent and discriminant validity, and metric invariance by sex—support the conceptualization of emotional trust in AI as a multidimensional construct that is empirically distinguishable from the cognitive or functional forms of trust traditionally examined.

Descriptive analyses showed adequate distributions for the 24 items, with means around the mid-to- high end of the scale and skewness and kurtosis values within acceptable ranges for factor modeling. This suggests that emotional trust in AI agents is not marginal in the studied university population, but rather a phenomenon present in everyday experience with generative tools. These results are consistent with recent studies documenting the transition of AI from a technical-instrumental role to symbolic and affective functions in daily interaction (Perrig et al., 2023; Scharowski and Perrig, 2023).

Regarding the internal structure, exploratory factor analysis showed that, although parallel analysis initially pointed to two factors, the MINRES solution with oblimin rotation revealed a three- dimensional structure in which Affective Recognition and Symbolic Bonding emerged as a highly correlated joint factor, while Perceived Authenticity and Emotional Validation formed distinct factors. This pattern is consistent with what is observed in affective scales with strongly interdependent components, where factors can be merged into an EFA due to their high covariation (Fabrigar et al., 1999; Volpato et al., 2025).

Confirmatory factor analysis, for its part, allowed for testing the originally proposed theoretical model and showed that a four-factor structure provides an adequate overall fit, with high standardized loadings across items and CFI, TLI, and SRMR values within recommended ranges. Although the robust RMSEA was in the moderate range, the convergence of the remaining indices supports the plausibility of the proposed model.

However, these results should be interpreted in conjunction with the exploratory factor analysis, which indicated a strong empirical overlap between Affective Recognition and Symbolic Bonding. Taken together, the EFA and CFA findings suggest that emotional trust in AI may be best understood as a set of closely related affective dimensions embedded within a broader emotional process, rather than as fully independent factors. This interpretation is consistent with affective measurement models in which theoretically distinct components exhibit substantial empirical interdependence.

This shift toward the emotional dimension of trust is especially relevant in light of recent findings on positive interaction with AI. The AI-Interaction Positivity Scale (AI-IPS) has demonstrated that positive emotions experienced during interaction with AI systems are associated with both globally favorable attitudes toward AI and higher levels of trust in tools like ChatGPT, as well as with a moderate increase in life satisfaction (Montag and Elhai, 2025). Our study aligns with this line of research by showing that the four factors of the AI-Interaction Positivity Scale (AI-IPS) exhibit high internal consistency (α and ω between 0.91 and 0.95) and average variances extracted (AVE) clearly exceeding 0.50, indicating that the emotional dimensions of trust constitute well-defined constructs and are not merely epiphenomena of perceived utility. In this sense, the AI-Interaction Positivity Scale offers a necessary complement to metrics focused on functional trust, allowing us to capture the affective quality of the human-AI relationship.

The moderate to high correlations observed between CEIA factors (*r* ≈ 0.65–0.90), together with elevated yet acceptable HTMT values, suggest the presence of an underlying emotional macroprocess rather than fully independent affective components. This pattern indicates that perceptions of authenticity, emotional validation, affective recognition, and symbolic bonding tend to co-occur within users' emotional experiences of human–AI interaction, reflecting a tightly integrated affective system. Importantly, similar configurations have been explicitly reported in recent studies on socio-emotional attachment to chatbots, where affective dimensions such as emotional support, perceived closeness, and relational bonding exhibit strong interdependence while remaining conceptually distinguishable (Bhat, 2025; Riley and Dixon, 2024; Yao et al., 2024).

From a broader interpretive perspective, these correlations raise relevant questions about the role of dispositional trust toward AI and usage-related factors in shaping affective responses. It is plausible that individuals with a generally negative or skeptical orientation toward AI may report lower levels of emotional trust across CEIA dimensions, even when they engage with generative systems frequently for instrumental purposes. Conversely, frequent users may develop higher affective trust through repeated exposure and positive interaction experiences, reinforcing emotional validation and symbolic bonding. Although dispositional trust and frequency of AI use were not explicitly modeled in the present study, future research should examine these variables as potential moderators or covariates to clarify how reliance, prior attitudes, and affective states jointly contribute to the formation of emotional trust in AI systems.

From a substantive perspective, our results align with evidence showing that many users turn to conversational agents not only for academic or informational tasks, but also to express sadness, depression, or personal concerns in culturally diverse contexts, perceiving AI as a safe space for emotional self-disclosure (Huynh and Aichner, 2025; Volpato et al., 2025). Similarly, qualitative studies with companion chatbots have documented that these agents can provide companionship, emotional containment, and even some informational and evaluative support, without offering tangible support (Zhang et al., 2025). In this scenario, the CEIA provides an empirical tool to quantify the extent to which these experiences of emotional support translate into stable affective trust in AI systems, and not merely into instrumental or episodic use of them.

The invariance analysis demonstrated metric equivalence between men and women, indicating that the CEIA measures emotional trust in AI in a structurally comparable manner across sexes and allowing for meaningful comparisons of relationships among variables. This finding is consistent with prior psychometric research on trust and affective constructs, where configural and metric invariance are commonly achieved across gender groups, reflecting shared conceptual understanding of the measured dimensions (Putnick and Bornstein, 2016; Milfont and Fischer, 2010).

However, only partial scalar invariance was supported, suggesting that some item thresholds may differ between men and women. Such patterns have been frequently reported in affective and relational scales and are often attributed to systematic differences in emotional expression, socialization processes, or response styles rather than to measurement bias *per se*. In the context of emotional trust in AI, it is plausible that men and women differ in the intensity with which they endorse affective items related to emotional validation or symbolic bonding, even when the underlying latent construct is comparable. Consequently, comparisons of latent means should be interpreted with caution. Future research should examine measurement invariance across additional grouping variables—such as intensity and purpose of AI use, academic discipline, or socioeconomic background—to further clarify how contextual and dispositional factors shape emotional trust in AI systems.

### 4.1 Theoretical and practical implications

On a practical level, having a robust measure of emotional trust has direct implications for interface design and institutional policies governing the use of generative AI in educational and mental health contexts. For example, universities could use the CEIA to assess whether specific design features—such as empathetic language, personalized feedback, or emotionally responsive prompts—are associated with higher levels of affective recognition or symbolic bonding. Prior research has shown that emotionally positive interactions with AI systems are linked to more favorable attitudes toward AI and higher levels of trust (Montag and Elhai, 2025), suggesting that interface characteristics may shape users' affective engagement. However, elevated scores in these dimensions may also indicate the need for safeguards to prevent excessive emotional reliance, particularly in contexts where AI begins to function as a source of emotional support (Perrig et al., 2023).

In educational settings, CEIA scores could be integrated into AI governance frameworks to monitor how students emotionally relate to generative tools beyond academic performance. For instance, students reporting high emotional validation and symbolic bonding but low perceived authenticity may benefit from guidance aimed at maintaining critical distance while using AI for emotional support. This distinction is especially relevant given recent warnings that traditional trust measures often blur the boundaries between trust, dependence, and reliance in human–AI interaction (Visser et al., 2025). When combined with measures of interaction positivity such as the AI-Interaction Positivity Scale (AI-IPS), the CEIA could help identify user profiles particularly prone to developing strong affective ties with AI systems (Montag and Elhai, 2025).

Furthermore, the scale offers concrete applications for psychoeducational interventions and AI literacy programs. Training modules could incorporate reflective activities based on CEIA dimensions to help students recognize how emotional trust in AI develops, differentiate between instrumental assistance and emotional substitution, and regulate their affective engagement. In applied contexts such as university counseling services, the CEIA could function as a screening or monitoring tool to support preventative strategies aimed at fostering healthy human–AI interaction without undermining meaningful interpersonal relationships, consistent with emerging evidence on socio-emotional attachment to conversational agents (Perrig et al., 2023; Volpato et al., 2025).

### 4.2 Limitations and future lines of research

This study has several limitations. First, it employed a cross-sectional design with a convenience sample of Peruvian university students; therefore, it is not possible to infer causal relationships or generalize the findings to different populations or diverse cultural contexts. Given that the affective relationship with AI can vary according to cultural, socioeconomic, and generational factors, it is necessary to replicate the CEIA validation in international samples and with users of different educational levels.

Second, the instrument's validity was primarily assessed in terms of internal structure, consistency, and convergent/discriminant validity based on the factor matrix. Future research should examine convergent and criterion validity with established AI confidence scales (e.g., S-TIAS, TXAI), interaction positivity measures (AI-IPS), and indicators of psychological wellbeing or distress to determine whether emotional confidence predicts attitudes, usage patterns, and relevant outcomes in mental health and academic functioning.

Third, although metric invariance by sex was supported, the evidence for scalar invariance was partial, and the most restrictive models showed some instability in certain parameters. This suggests caution when comparing latent means between men and women and points to the need to explore invariance in other criteria (such as intensity of AI use, fields of study, or socioeconomic level), as well as to use partial invariance models where appropriate.

Finally, the CEIA relies on self-reports, with the inherent limitations of social desirability bias and introspection about emotional states. Future studies could complement the CEIA with qualitative methods, analyses of real-life conversations with AI, and behavioral measures of use, as well as longitudinal designs that allow us to examine whether emotional trust predicts changes in AI use or progressive shifts from human support sources to artificial agents.

Taken together, the results suggest that the CEIA constitutes a solid and timely contribution to psychometrics applied to AI, by operationalizing an emotional dimension of trust that is gaining relevance in everyday life and in educational settings, but which until now had been little studied.

## 5 Conclusion

The results of the study indicate that the Emotional Trust in Artificial Intelligence Scale (CEIA) is a valid, reliable, and structurally sound instrument for assessing the affective dimension of human-AI interaction in university students. The scale showed a four-dimensional model consistent with the theoretical framework and empirically supported by confirmatory factor analysis, as well as adequate convergent and discriminant validity indices. Furthermore, the metric invariance by sex suggests that comparisons between men and women can be made without bias in the item-factor relationship, although scalar invariance was only partially supported, which warrants caution in interpreting comparisons of latent means.

Given that emotional trust in AI can be associated with academic, affective, and relational processes—including the perception of emotional support, the attribution of authenticity, and the formation of symbolic bonds with artificial agents—having a psychometrically robust measure is key to understanding this emerging phenomenon. The findings highlight the need to integrate interdisciplinary approaches that bring together psychology, cognitive science, and human-computer interaction studies to explore how forms of affective trust are configured, maintained, and transformed in everyday contexts.

Overall, this study provides a psychometrically robust instrument for assessing emotional trust in artificial intelligence, a dimension that has remained largely underrepresented in existing trust frameworks. By operationalizing affective components such as emotional validation, perceived authenticity, affective recognition, and symbolic bonding, the CEIA advances current understanding of human–AI interaction beyond purely cognitive or functional perspectives. The findings underscore the relevance of emotional trust as a meaningful and measurable construct within contemporary educational contexts, offering a validated tool to support both empirical research and informed decision-making regarding the integration of generative AI systems in human-centered environments.

## Data Availability

The datasets presented in this study can be found in online repositories. The names of the repository/repositories and accession number(s) can be found in the article/Supplementary material.

## References

[B1] Aiken L. R. (1980). Content validity and reliability of single items or questionnaires. Educ. Psychol. Meas. 40, 955–959. doi: 10.1177/001316448004000419

[B2] Alonso Moral J. M. (2025). Tutorial: fundamentals of (and tools for) trustworthy artificial intelligence in smart health. Int. Conf. EDemo. EGov. ICEDEG 2025, 11–13. doi: 10.1109/ICEDEG65568.2025.11081632

[B3] Ato M. López J. J. Benavente A. (2013). Un sistema de clasificación de los diseños de investigación en psicología. Ann. Psychol. 29, 1038–1059. doi: 10.6018/analesps.29.3.178511

[B4] Bhat M. (2025). “How dynamic vs. static presentation shapes user perception and emotional connection to text-based AI,” in Proceedings of the 30th International Conference on Intelligent User Interfaces (IUI'25) [New York, NY: Association for Computing Machinery (ACM)], 846–860. doi: 10.1145/3708359.3712131

[B5] Brown T. A. (2015). Confirmatory Factor Analysis for Applied Research, 2nd Edn. New York, NY: The Guilford Press.

[B6] Chen F. F. (2007). Sensitivity of goodness of fit indexes to lack of measurement invariance. Struct. Eq. Model.: Multidiscipl. J. 14, 464–504. doi: 10.1080/10705510701301834

[B7] Chin H. Song H. Baek G. Shin M. Jung C. Cha M. . (2023). The potential of chatbots for emotional support and promoting mental well-being in different cultures: mixed methods study. J. Med. Internet Res. 25:e51712. doi: 10.2196/51712 37862063 PMC10625083

[B8] Comrey A. L. Lee H. B. (1992). A First Course in Factor Analysis, 2nd Edn. Lawrence Erlbaum Associates.

[B9] Dorton S. L. Harper S. B. (2022). Self-repairing and/or buoyant trust in artificial intelligence. Proc. Hum. Fact. Ergon. Soc. 66, 162–166. doi: 10.1177/1071181322661098

[B10] Epley N. Waytz A. Cacioppo J. T. (2007). On seeing human: a three-factor theory of anthropomorphism. Psychol. Rev. 114, 864–886. doi: 10.1037/0033-295X.114.4.864 17907867

[B11] Fabrigar L. R. MacCallum R. C. Wegener D. T. Strahan E. J. (1999). Evaluating the use of exploratory factor analysis in psychological research. Psychol. Methods 4, 272–299. doi: 10.1037/1082-989X.4.3.272

[B12] Finney S. (2013). Nonnormal and Categorical Data in Structural Equation Modeling. Selected Works. Available online at: https://commons.lib.jmu.edu/selectedworks/153 (Accessed October 10, 2025).

[B13] Flora D. B. Curran P. J. (2004). An empirical evaluation of alternative methods of estimation for confirmatory factor analysis with ordinal data. Psychol. Methods 9, 466–491. doi: 10.1037/1082-989X.9.4.466 15598100 PMC3153362

[B14] Fornell C. Larcker D. F. (1981). Evaluating structural equation models with unobservable variables and measurement error. J. Market. Res. 18, 39–50. doi: 10.1177/002224378101800104

[B15] Henseler J. Ringle C. M. Sarstedt M. (2014). A new criterion for assessing discriminant validity in variance-based structural equation modeling. J. Acad. Market. Sci. 43, 115–135. doi: 10.1007/s11747-014-0403-8

[B16] Henseler J. Ringle C. M. Sarstedt M. (2015). A new criterion for assessing discriminant validity in variance-based structural equation modeling. J. Acad. Market. Sci. 43, 115–135. doi: 10.1007/s11747-014-0403-8

[B17] Horton D. Wohl R. R. (1956). Mass communication and para-social interaction. Psychiatry 19, 215–229. doi: 10.1080/00332747.1956.11023049 13359569

[B18] Hu L. T. Bentler P. M. (1999). Cutoff criteria for fit indexes in covariance structure analysis: conventional criteria versus new alternatives. Struct. Eq. Model.: Multidiscipl. J. 6, 1–55. doi: 10.1080/10705519909540118

[B19] Huynh M. T. Aichner T. (2025). In generative artificial intelligence we trust: unpacking determinants and outcomes for cognitive trust. AI Soc. 40, 5849–5869. doi: 10.1007/s00146-025-02378-8

[B20] Jiang W. Li D. Liu C. (2025). Understanding dimensions of trust in AI through quantitative cognition: implications for human-AI collaboration. PLoS ONE 20:e0326558. doi: 10.1371/journal.pone.0326558 40601655 PMC12221052

[B21] Kline R. B. (2016). Principles and Practice of Structural Equation Modeling, 4th Edn. New York, NY: The Guilford Press. Available online at: https://psycnet.apa.org/record/2015-56948-000

[B22] Kolomaznik M. Petrik V. Slama M. Jurik V. (2024). The role of socio-emotional attributes in enhancing human-AI collaboration. Front. Psychol. 15;1369957. doi: 10.3389/fpsyg.2024.1369957 39474095 PMC11518774

[B23] Kwilinski A. Lyulyov O. Pimonenko T. (2025). “Emotion recognition of artificial intelligence for enhancing consumer trust in the galvanic skin response,” in New Challenges of the Global Economy for Business Management: EEEU 2024. Springer Proceedings in Business and Economics, eds. S. Kot, B. Khalid, and A. ul Haque (Singapore: Springer). doi: 10.1007/978-981-96-4116-1_107

[B24] Liu W. Zhang S. Zhang T. Gu Q. Han W. Zhu Y. (2024). The AI empathy effect: a mechanism of emotional contagion. J. Hosp. Tour. Technol. 15, 703–734. doi: 10.1080/19368623.2024.2315954

[B25] MacCallum R. C. Widaman K. F. Zhang S. Hong S. (1999). Sample size in factor analysis. Psychological MethodsPsychol. Methods 4, 84–99. doi: 10.1037/1082-989X.4.1.84

[B26] Martín-Moncunill D. García Laredo E. Carlos Nieves J. (2024). POTDAI: a tool to evaluate the perceived operational trust degree in artificial intelligence systems. IEEE Access 12, 133097–133109. doi: 10.1109/ACCESS.2024.3454061

[B27] McDonald R. P. (2013). Test Theory: A Unified Treatment. New York, NY: Psychology Press. doi: 10.4324/9781410601087

[B28] McGrath M. J. Lack O. Tisch J. Duenser A. (2025). Measuring trust in artificial intelligence: validation of an established scale and its short form. Front. Artif. Intell. 8:1582880. doi: 10.3389/frai.2025.1582880 40416549 PMC12098057

[B29] Milfont T. L. Fischer R. (2010). Testing measurement invariance across groups: applications in cross-cultural research. Int. J. Psychol. Res. 3, 111–121. doi: 10.21500/20112084.857

[B30] Mokkink L. B. Terwee C. B. Patrick D. L. Alonso J. Stratford P. W. Knol D. L. . (2010). The COSMIN checklist for assessing the methodological quality of studies on measurement properties of health status measurement instruments: an international Delphi study. Qual. Life Res. 19, 539–549. doi: 10.1007/s11136-010-9606-8 20169472 PMC2852520

[B31] Montag C. Elhai J. D. (2025). Introduction of the AI-interaction positivity scale and its relations to satisfaction with life and trust in ChatGPT. Comput. Human Behav. 172:108705. doi: 10.1016/j.chb.2025.108705

[B32] Morales-García W. C. Sairitupa-Sanchez L. Z. Morales-García S. B. Morales-García M. (2024). Development and validation of a scale for dependence on artificial intelligence in university students. Front. Educ. 9:1323898. doi: 10.3389/feduc.2024.1323898

[B33] Nass C. Moon Y. (2000). Machines and mindlessness: social responses to computers. J. Soc. Issues 56, 81–103. doi: 10.1111/0022-4537.00153

[B34] Nunnally J. C. Bernstein I. H. (1994). Psychometric Theory, 3rd Edn. McGraw-Hill.

[B35] Penfield R. D. Giacobbi P. R. (2004). Applying a score confidence interval to Aiken's item content-relevance index. Meas. Phys. Educ. Exerc. Sci. 8, 213–225. doi: 10.1207/s15327841mpee0804_3

[B36] Perrig S. A. C. Scharowski N. Brühlmann F. (2023). “Trust issues with trust scales: examining the psychometric quality of trust measures in the context of AI,” in Extended Abstracts of the 2023 CHI Conference on Human Factors in Computing Systems (Article 297). [New York, NY: Association for Computing Machinery (ACM)]. doi: 10.1145/3544549.3585808

[B37] Putnick D. L. Bornstein M. H. (2016). Measurement invariance conventions and reporting: the state of the art and future directions for psychological research. Dev. Rev. 41, 71–90. doi: 10.1016/j.dr.2016.06.004 27942093 PMC5145197

[B38] R Core Team (2023). R: The R Project for Statistical Computing. Vienna: R Foundation for Statistical Computing. Available online at: https://www.r-project.org/ (Accessed October 10, 2025).

[B39] Riley B. K. Dixon A. (2024). Emotional and cognitive trust in artificial intelligence: a framework for identifying research opportunities. Curr. Opin. Psychol. 58:101833. doi: 10.1016/j.copsyc.2024.101833 38991423

[B40] Rogers C. R. (1961). On Becoming a Person: A Therapist's View of Psychotherapy. Houghton Mifflin.

[B41] Rogers C. R. (2012). On Becoming a Person: A Therapist's View of Psychotherapy. Boston, MA: Houghton Mifflin Harcourt.

[B42] Scharowski N. Perrig S. A. C. (2023). Distrust in (X)AI–Measurement artifact or distinct construct? arXiv preprint arXiv:2303.1649. Available online at: https://arxiv.org/pdf/2303.16495 (Accessed October 10, 2025).

[B43] Schumacker R. E. Lomax R. G. (2015). A Beginner's Guide to Structural Equation Modeling, 4th Edn. New York, NY: Routledge. doi: 10.4324/9781315749105

[B44] Shang R. Hsieh G. Shah C. (2024). Trusting your AI agent emotionally and cognitively: development and validation of a semantic differential scale for AI trust. Proc. AAAI/ACM Conf. AI, Ethics Soc. 7, 1343–1356. doi: 10.1609/aies.v7i1.31728

[B45] Sun Y. Xu C. Xu H. (2024). Social identity in trusting artificial intelligence agents: evidence from lab and online experiments. Manag. Decis. Econ. 45, 5899–5916. doi: 10.1002/mde.4361

[B46] Ta V. Griffith C. Boatfield C. Wang X. Civitello M. Bader H. . (2020). User experiences of social support from companion chatbots in everyday contexts: thematic analysis. J. Med. Internet Res. 22:e16235. doi: 10.2196/16235 32141837 PMC7084290

[B47] Torres-Gastelú C. A. Torres-Real C. (2025). Validación de una escala sobre la percepción de la Inteligencia Artificial en la educación superior. Ciencia Latina Rev. Científ. Multidiscipl. 9, 5706–5725. doi: 10.37811/cl_rcm.v9i2.17324

[B48] VandenBos G. R. (ed.). (2015). APA Dictionary of Psychology, 2nd Edn. American Psychological Association. doi: 10.1037/14646-000

[B49] Visser R. Peters T. M. Scharlau I. Hammer B. (2025). Trust, distrust, and appropriate reliance in (X)AI: a conceptual clarification of user trust and survey of its empirical evaluation. Cogn. Syst. Res. 91:101357. doi: 10.1016/j.cogsys.2025.101357

[B50] Volpato R. DeBruine L. Stumpf S. (2025). Trusting emotional support from generative artificial intelligence: a conceptual review. Comput. Hum. Behav.: Artif. Hum. 5;100195. doi: 10.1016/j.chbah.2025.100195

[B51] Wang P. Yin K. Tian M. Zheng Y. Wu H. Zhou C. . (2025). A validation of the human-generative artificial intelligence trust scale. Int. J. Hum. Comput. Interact. doi: 10.1080/10447318.2025.2542881

[B52] Yao D. Holopainen J. Laukkanen T. (2024). “Human-AI interaction-Is it trust or emotions that mediates behavioral intentions?,” in Proceedings of the Annual Hawaii International Conference on System Sciences. Available online at: https://www.scopus.com/pages/publications/85199785733?origin=document-preview-flyout (Accessed October 10, 2025).

[B53] Zhang X. Li Z. Zhang M. Yin M. Yang Z. Gao D. . (2025). Exploring artificial intelligence (AI) Chatbot usage behaviors and their association with mental health outcomes in Chinese university students. J. Affect. Disord. 380, 394–400. doi: 10.1016/j.jad.2025.03.141 40147615

