# Supplementary material for: Construction and validation of the Emotional Trust in Artificial Intelligence Scale (CEIA)
Source: Front Psychol. 2026 Mar 12;17:1755160. doi: 10.3389/fpsyg.2026.1755160 (PMC13017824; doi:10.3389/fpsyg.2026.1755160)
Supplement: Supplementary file 1 [file Supplementary_file_1.docx]

Supplementary Material

# Supplementary Data

1. **Supplementary Material A**
2. **Emotional Trust in Artificial Intelligence Scale (CEIA)**

**Construcción y validación de la Escala de Confianza Emocional en la Inteligencia Artificial (CEIA) en estudiantes universitarios**

1. **Descripción del constructo**

La **Confianza Emocional en la Inteligencia Artificial (CEIA)** se define como el grado en que una persona percibe que un sistema de inteligencia artificial es capaz de comprender, validar y responder de manera coherente y significativa a sus estados emocionales, generando experiencias de autenticidad percibida, reconocimiento afectivo y vínculo simbólico.

El constructo se organiza en **cuatro dimensiones teóricas**:

- **Autenticidad percibida (AP):**
  Grado en que el usuario percibe que la IA expresa respuestas emocionales de manera genuina y coherente, y no meramente mecánica o programada.
- **Validación emocional (VE):**
  Grado en que el usuario percibe que la IA reconoce, comprende y acepta sus emociones, generando sensación de acompañamiento, alivio y aceptación emocional.
- **Reconocimiento afectivo (RA):**
  Capacidad percibida de la IA para identificar correctamente el tono emocional o estado afectivo del usuario y ajustar sus respuestas en consecuencia.
- **Vínculo simbólico (VS):**
  Sentido de conexión emocional significativa o pertenencia que el usuario establece con la IA, percibiéndola como una compañía simbólica o entidad emocionalmente relevante, aun reconociendo su naturaleza no humana.

1. **Instrucciones para los participantes**

A continuación, encontrará una serie de afirmaciones sobre su interacción con sistemas de inteligencia artificial (por ejemplo, asistentes virtuales tipo ChatGPT, chatbots de ayuda, aplicaciones de bienestar).

Indique en qué medida está de acuerdo con cada afirmación según su experiencia personal. Responda con sinceridad; **no existen respuestas correctas o incorrectas**.

1. **Escala de respuesta (Likert de 5 puntos)**

1 = Totalmente en desacuerdo
2 = En desacuerdo
3 = Ni de acuerdo ni en desacuerdo
4 = De acuerdo
5 = Totalmente de acuerdo

1. **Ítems de la Escala de Confianza Emocional en la IA (CEIA)**
2. **Dimensión A — Autenticidad percibida (AP)**

AP1. Las respuestas de la IA me resultan sinceras y coherentes con lo que le comento.
AP2. La IA responde de forma genuina, no solo con datos fríos.
AP3. Cuando interactúo con la IA, siento que su respuesta refleja un intento real de entendimiento.
AP4. Las respuestas de la IA son coherentes con el tono emocional de la conversación.
AP5. La IA ajusta sus respuestas de forma auténtica según lo que expreso.
AP6. La comunicación de la IA me parece honesta y auténtica.

1. **Dimensión B — Validación emocional (VE)**

VE1. Siento que la IA reconoce mis sentimientos cuando interactúo con ella.
VE2. Las respuestas de la IA me hacen sentir comprendido/a en lo emocional.
VE3. Las respuestas de la IA me hacen sentir acompañado/a emocionalmente.
VE4. La IA ofrece comentarios que me ayudan a aceptar o validar lo que siento.
VE5. Sentir que la IA valida mis emociones me genera calma o alivio.
VE6. Considero que la IA puede ser útil para expresar emociones y sentirse comprendido/a.

1. **Dimensión C — Reconocimiento afectivo (RA)**

RA1. La IA suele identificar correctamente el tono emocional de mis mensajes.
RA2. La IA detecta cuándo estoy triste, preocupado/a o contento/a.
RA3. Las respuestas de la IA cambian según mi estado de ánimo.
RA4. La IA demuestra haber comprendido mi estado emocional.
RA5. Confío en que la IA puede reconocer mis emociones incluso si no las explicito por completo.
RA6. La IA responde de forma apropiada cuando expreso angustia o estrés.

1. **Dimensión D — Vínculo simbólico (VS)**

VS1. A veces siento una conexión personal con la IA tras varias interacciones.
VS2. Considero a la IA como una compañía con la que puedo conversar sobre asuntos personales.
VS3. Me resulta cómodo/a recurrir a la IA cuando necesito desahogarme.
VS4. En ocasiones me resulta más fácil expresarme con la IA que con otras personas.
VS5. Siento que la relación con la IA tiene valor emocional para mí, aunque sé que no es humana.
VS6. Me gustaría que la IA continuara disponible para conversaciones futuras sobre temas emocionales.

1. **Puntuación e interpretación**

- **Puntuaciones por dimensión:** se obtiene calculando el promedio (o suma) de los ítems correspondientes a cada subescala (AP, VE, RA, VS).
- **Puntuación total CEIA:** se obtiene promediando (o sumando) los 24 ítems.
- **Interpretación:** puntuaciones más altas indican mayores niveles de confianza emocional en la inteligencia artificial.
  Dada la alta consistencia interna y la fuerte interrelación entre dimensiones, la escala puede interpretarse tanto a nivel **dimensional** como mediante un **puntaje global** de confianza emocional.

**
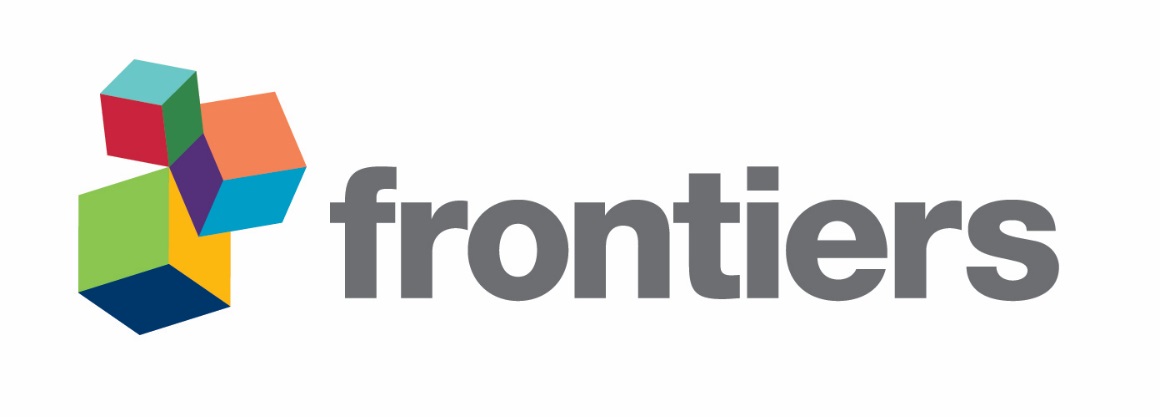
**
